# Phylogenetic Analysis and Substitution Rate Estimation of Colonial Volvocine Algae Based on Mitochondrial Genomes

**DOI:** 10.3390/genes11010115

**Published:** 2020-01-20

**Authors:** Yuxin Hu, Weiyue Xing, Zhengyu Hu, Guoxiang Liu

**Affiliations:** 1 Key Laboratory of Algal Biology, Institute of Hydrobiology, Chinese Academy of Sciences, Wuhan 430072, China; 2 School of Life Sciences, University of Chinese Academy of Sciences, Beijing 100049, China; 3 State Key Laboratory of Freshwater Ecology and Biotechnology, Institute of Hydrobiology, Chinese Academy of Sciences, Wuhan 430072, China

**Keywords:** colonial volvocine algae, dN/dS ratio, mitochondrial genome, phylogenetic analysis, substitution rates

## Abstract

We sequenced the mitochondrial genome of six colonial volvocine algae, namely: *Pandorina morum*, *Pandorina colemaniae*, *Volvulina compacta*, *Colemanosphaera angeleri*, *Colemanosphaera charkowiensi*, and *Yamagishiella unicocca*. Previous studies have typically reconstructed the phylogenetic relationship between colonial volvocine algae based on chloroplast or nuclear genes. Here, we explore the validity of phylogenetic analysis based on mitochondrial protein-coding genes. We found phylogenetic incongruence of the genera *Yamagishiella* and *Colemanosphaera*. In *Yamagishiella*, the stochastic error and linkage group formed by the mitochondrial protein-coding genes prevent phylogenetic analyses from reflecting the true relationship. In *Colemanosphaera*, a different reconstruction approach revealed a different phylogenetic relationship. This incongruence may be because of the influence of biological factors, such as incomplete lineage sorting or horizontal gene transfer. We also analyzed the substitution rates in the mitochondrial and chloroplast genomes between colonial volvocine algae. Our results showed that all volvocine species showed significantly higher substitution rates for the mitochondrial genome compared with the chloroplast genome. The nonsynonymous substitution (dN)/synonymous substitution (dS) ratio is similar in the genomes of both organelles in most volvocine species, suggesting that the two counterparts are under a similar selection pressure. We also identified a few chloroplast protein-coding genes that showed high dN/dS ratios in some species, resulting in a significant dN/dS ratio difference between the mitochondrial and chloroplast genomes.

## 1. Introduction

With the application of next-generation sequencing technology, an increasing number of mitochondrial genomes (mtDNA) have been sequenced in recent years, but only a small number of mtDNA genomes are available in colonial volvocine algae (Chlorophyta, Chlamydomonadales). For colonial volvocine algae, the first mtDNA genome sequence was reported in 1989. Moore and Coleman [1] constructed a physical restriction map of mtDNA from the species *Pandorina morum*. This species has a linear 20 kb genome, but the sequence of this mtDNA is not available online. In 2010, the second mtDNA of colonial volvocine algae was sequenced. Smith and Lee [2] reported a circular mtDNA genome of *Volvox carteri*, and proposed a mutational-hazard hypothesis. In 2013, Hamaji et al. [3] presented the complete mtDNA of the species *Gonium pectoral.* They, compared the circular mtDNA of *Gonium pectorale* with *Volvox carteri* and other unicellular species. Their comparison raised questions about the origin of linear mtDNA in Volvocales. Meanwhile, Smith et al. [4] sequenced the mtDNA of *Pleodorina starrii* and explored the relationship between organelle genome complexity and organism size. The mtDNA genome of *Tetrabaena socialis* was the next to be sequenced. Featherston et al. [5] showed a circular-mapping mtDNA architecture at the origin of the colonial volvocine algae. These different mtDNA architectures prompted the community’s interest in the origin and evolution of mtDNA conformation in colonial volvocine algae. To answer this question, Hamaji et al. [6] sequenced the mtDNA of *Yamagishiella unicocca* and *Eudorina* sp. Analysing all of the available mtDNA of colonial volvocine algae and several unicellular species at that time, they proposed at least three separate shifts in mtDNA architecture in the Reinhardtinia clade. Finally, in 2019, we presented two mtDNA genomes in the genus *Eudorina* [7]. We analysed the mtDNA organization and offered information to discover the composition of mtDNA in colonial volvocine algae. To the best of our knowledge, these mtDNA genomes are only available in colonial volvocine algae.

For colonial volvocine algae, previous studies have mainly focused on the conformation of mtDNA, and few have reported phylogenetic analyses based on mtDNA protein-coding genes in this group of algae. Phylogeny based on mtDNA has been widely used in different types of species, such as fish, parasites, brown algae, red algae, and green algae [8,9,10,11,12]. The phylogenetic analyses among colonial volvocine algae in previous studies were mainly based on genes such as the internal transcribed spacer (ITS), the large subunit of the ribulose-1,5-bisphosphate carboxylase/oxygenase (rbcL) gene, five chloroplast protein-coding genes, and the chloroplast genome [7,13,14,15,16,17,18,19,20,21,22,23,24,25]. Thus, in this study, we try to explore whether the mtDNA protein-coding genes are suitable for phylogenetic reconstruction in colonial volvocine algae.

We have presented several chloroplast genomes (ptDNA) of colonial volvocine algae in our previous studies [7,25]. Together with the mtDNA sequenced in this study, we are able to compare the substitution rates between their mtDNA and ptDNA. mtDNA and ptDNA are very similar in many respects. Both genomes are housed in energy-producing organelles, are highly reduced, and are dependent on nuclear-encoded and organelle-targeted proteins [26]. Although mtDNA and ptDNA have many similar characteristics, they showed different substitution rates in different groups of species, such as seed plants and algae [27,28,29,30]. The different organelle substitution rates may be associated with the nature and accuracy of organelle-targeted DNA maintenance machineries [31], and the number of genes that an organelle possesses [26]. In this study, we compared the substitution rates between mtDNA and ptDNA among colonial volvocine algae, so as to offer more information about the substitution rates in organelle genomes.

## 2. Materials and Methods 

### 2.1. Cultures

Six colonial volvocine algae were obtained from the Culture Collection of Freshwater Algae at the Institute of Hydrobiology, Chinese Academy of Sciences. The strain numbers were as follows: *Pandorina morum* (strain FACHB-2362), *Pandorina colemaniae* (strain FACHB-2361), *Volvulina compacta* (strain FACHB-2337), *Colemanosphaera angeleri* (strain FACHB-2363), *Colemanosphaera charkowiensis* (strain FACHB-2326), and *Yamagishiella unicocca* (strain FACHB-2364). All of the algae were cultured in a BG11 medium, and the cultures were grown at 20–25 °C under a 14 h light:10 h dark schedule under cool-white fluorescent lamps, at an intensity of 1000–2000 lux.

### 2.2. Sequencing, Assembly, and Annotation

All of the strains were sequenced and assembled as described previously [7,25]. mtDNA was initially annotated using the MITOchondrial genome annotation Server (MITOS) [32]. Protein-coding and ribosomal RNA (rRNA) genes were further updated using Basic Local Alignment Search Tool (BLAST+) [33], with genes from the available colonial volvocine mtDNA. The transfer RNA (tRNA) genes were redetected using tRNAscan-SE [34]. All of the mitochondrial genome sequences were submitted to GenBank; the accession numbers are listed in Table 1. The synteny comparison was visualized using progressiveMauve [35].

### 2.3. Phylogenetic Analysis

The nucleotide sequences of the protein-coding genes were used for the phylogenetic analysis. Each gene was aligned using MAFFT v7.394 (a multiple sequence alignment program) [36], and the alignments of all of the genes were converted into a codon alignment using TranslatorX [37]. The ambiguously aligned regions were excluded using trimAl v1.2 [38], with the option -gt = 1 (gapthreshold). The third codon positions of each gene were removed because of substitution saturation.

We used supermatrix and coalescent-based analyses to construct the phylogenetic tree. For the supermatrix analysis, Phyutility [39] was used to concatenate all of the genes. The first and second codons of each gene were considered as different partitions. The selections of the evolutionary model and the partition of each gene were performed using PartitionFinder 2 [40]. The phylogenies were inferred using the maximum likelihood (ML) and Bayesian inference (BI) methods. The ML analyses were performed using Randomized Axelerated Maximum Likelihood program (RAxML) v8.2.10 [41]. A rapid bootstrap analysis with 1000 replicates of the dataset for ML was performed to estimate the statistical reliability. A Bayesian analysis was performed with MrBayes v3.2.6 [42]. A Markov chain Monte Carlo analysis was performed with four Markov chains (three heated and one cold) for 1,000,000 generations, with trees sampled every 1000 generations. Each time the diagnostics were calculated, a fixed number of samples (burnin = 1000) were discarded from the beginning of the chain. A stationary distribution was assumed when the average standard deviation of the split frequencies was less than 0.01. For the coalescent-based analyses, an ML analysis of each gene was performed in RAxML v8.2.10 [41] by conducting 1000 rapid bootstrap replicates with the General Time Reversible model with estimate of proportion of invariable sites and rate heterogeneity (GTRGAMMAI model). Then, the resulting best trees were used to infer the coalescence-based species tree phylogeny with Accurate Species TRee ALgorithm program (ASTRAL) v5.6.3 [43].

To examine the systematic errors, we used PAUP* version 4.0a166 for the minimum evolution and LogDet distances from the data, and coded both as standard nucleotides (NT-coding) and as purines and pyrimidines (A&G→R, C&T→Y, and RY-coding) [44]. We used MrBayes v3.2.6 as the covarion model for the data, as the other options were the same as the supermatrix approach.

### 2.4. Substitution Rate Estimation

After each gene was aligned and trimmed as performed in the phylogenetic analysis, synonymous and nonsynonymous substitutions were measured using the CODEML program of the phylogenetic analysis by maximum likelihood (PAML) v4.9 [45]. The ML model was used with the following options: runmode = −2 and CodonFreq = 2. Genes with synonymous substitution (dS) values greater than five were discarded from further analysis. Synonymous and nonsynonymous substitution values were averaged for all of the pairwise comparisons of each gene. Statistical analyses were implemented in R (http://www.R-project.org).

## 3. Results

### 3.1. Mitochondrial Genome of Colonial Volvocine Algae

The general features of all of the available mtDNA to date among the colonial volvocine algae are listed in Table 1. The size of mtDNA ranged from 15 to 35 kb. *Volvox carteri* is the most complex organism and possesses the largest mtDNA size. However, in all colonial species, there was no correlation between organism complexity and mtDNA size. The GC content in all of the mtDNA in the colonial volvocine species was less than 45%. The GC content ranged from 33% to 45%. With the exception of *Pandorina morum,* sequenced in this study, which has five scaffolds, the other species have complete mtDNA or only one scaffold. The number of protein coding genes ranges from 7 to 12. Only seven genes were found in all of the species, namely: *cob*, *cox1*, *nad1*, *nad2*, *nad4*, *nad5*, and *nad6*. These genes were used for our downstream analysis. The number of rRNA genes ranged from 3 to 12. The rRNA genes were composed of two types of genes, namely: *rrnL* and *rrnS*. Normally, *rrnL* contains eight fragments, and *rrnS* contains four fragments. We noticed that *Volvox carteri* has 13 rRNA genes, because it has two copies of *rrnL* fragment 8. For the tRNA genes, most species have three tRNA genes, namely: *trnM*, *trnW,* and *trnQ*. Only *Gonium pectorale* has one additional copy of *trnM*. *Volvulina compacta* has one extra *trnE* gene. We only annotated one tRNA gene for *Pandorina morum*.

**Figure 1:**
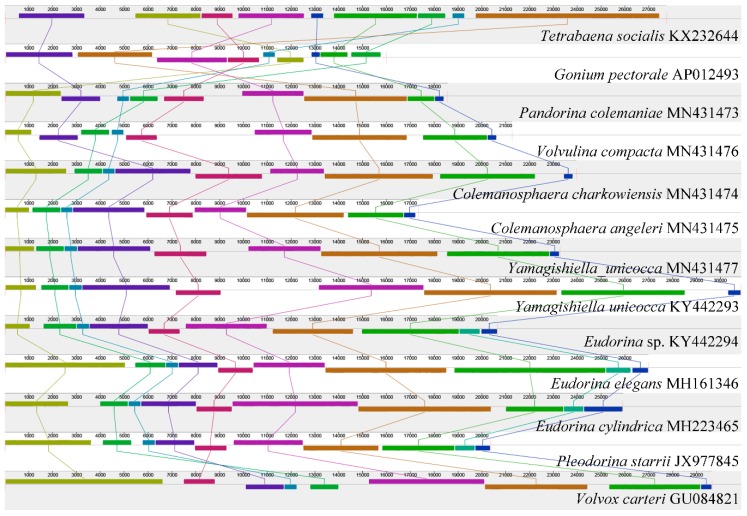
Synteny comparison of colonial volvocine algae organelle genomes based on the mitochondrial genome. The colored syntenic blocks are local collinear blocks; blocks above the center line indicate they are on the same strand, and blocks below the center line indicate they are on the opposite strand.

The mtDNA synteny analysis is shown in Figure 1. All of the mtDNA of the colonial volvocine algae currently available were used for the analysis (except *Pandorina morum,* which has five scaffolds), with 13 mtDNA in total (12 species from nine genera). For *Tetrabaena socialis* (belonging to the family Tetrabaenaceae), *Gonium pectorale* (belonging to the family Goniaceae), and *Pandorina colemaniae* (belonging to the family Volvocaceae), considerable amounts of rearrangement and inversion have occurred. These three species belong to different families; this result is consistent with our previous study [7]. For the different genera in the family Volvocaceae (except for *Tetrabaena socialis* and *Gonium pectorale*, all of the other species we used belong to the family Volvocaceae), most of the genera showed a highly conserved synteny relationship, with a few exceptions (among *Pandorina colemaniae*, *Volvulina compacta*, and *Colemanosphaera charkowiensis*; between *Pleodorina starrii* and *Volvox carteri*). Species belonging to the same genera or the same species did not show any rearrangement or reversal.

Overall, the mtDNA structure in colonial volvocine algae showed a high variation among the different families, little variation among different genera from the same family, and it is highly conserved for species in the same genera or for different strains belonging to the same species.

### 3.2. Phylogenetic Analysis

**Figure 2:**
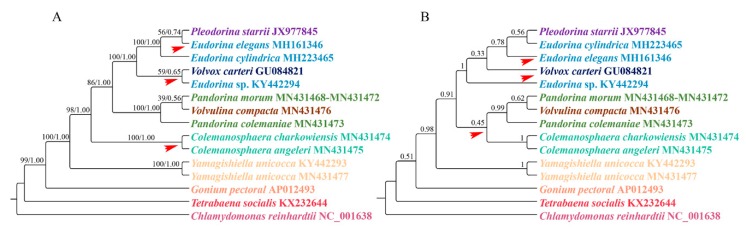
Phylogenetic tree of the colonial volvocine algae based on mitochondrial protein-coding genes. (**A**) Phylogenetic analysis based on the supermatrix approach. Numbers on the left and right side of the branches represent bootstrap values and Bayesian posterior probabilities respectively. (**B**) Phylogenetic analysis based on coalescent-based approach. The numbers at the branches represent the support values of ASTRAL. Each genus is indicated by a different color, with the red arrow indicating inconsistent branches.

For the supermatrix analysis, the phylogenetic trees inferred from the Bayesian and maximum likelihood analyses are the same. The ML tree was used to represent the results (Figure 2A). For the coalescent-based analysis, the resulting phylogenetic tree is presented in Figure 2B.

**Figure 3:**
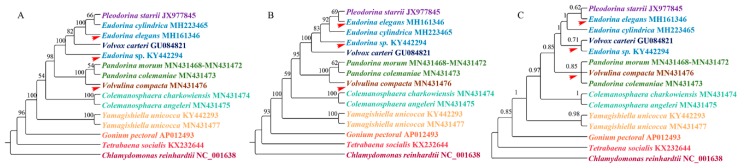
Bootstrap support from 1000 replicates for the phylogenetic analysis based on three different methods. (**A**) Phylogenetic analysis based on LogDet distances. (**B**) Phylogenetic analysis based on the RY-coding strategy. (**C**) Phylogenetic analysis based on the covarion model. Each genus is indicated by a different color, with a red arrow indicating inconsistent branches.

When we compared the supermatrix and coalescent-based analysis results, the two methods showed different topologies among species within the clade *Eudorina*/*Pleodorina*/*Volvox*. Notably, the phylogenetic position of the genus *Colemanosphaera*, based on the coalescent-based analysis, is consistent with Nozaki et al. [23], but this phylogenetic relationship is not supported by our supermatrix analysis. Our results showed that *Yamagishiella unicocca* formed a clade. This clade is sister to the clade formed by all of the other Volvocaceae species with high support values. This phylogenetic relationship is different from all previous studies [20,21,22,23]. In phylogenomics, systematic errors become stronger when multiple genes are combined into a supermatrix, thus resulting in an incorrect phylogenetic relationship with a high support value [47]. A systematic error is mainly due to a compositional signal, rate signal, and heterotachous signal [48]. The LogDet distance takes irregular A, C, G, and T compositions into consideration [49]. The RY-coding strategy can discard fast-evolving transitions and make phylogenetic reconstruction less susceptible to multiple hits between lineages [44,50]. The covarion model allows for the rate at a site to change over its evolutionary history [51]. Here, we used LogDet distances, the RY-coding strategy, and covarion model to exam the systematic error. The results are shown in Figure 3. For the genera *Colemanosphaera* and *Yamagishiella*, all of our methods showed the same phylogenetic relationship with the supermatrix approach. Thus, the systematic error was not severe enough to affect the phylogenetic results of these two genera. However, the three methods showed different phylogenetic relationships in clade *Eudorina*/*Pleodorina*/*Volvox* and clade *Pandorina*/*Volvulina*. Thus, our phylogenetic analyses were generally affected by the systematic errors.

### 3.3. Substitution Rate Estimation

In this study, we used the ML method implemented in PAML to calculate the synonymous and nonsynonymous substitution rate. This method is the most accurate method currently available for measuring substitution rates [45,52]. Substantial differences in synonymous substitutions were not noted among colonial species (Table 2). The average dS values for all of the species varied from 1.97 to 2.85 for mitochondrial genes, and from 0.78 to 1.31 for chloroplast genes. All of the species showed higher average dS values in the mtDNA. Among them, *Pandorina morum* showed the biggest difference, with the dS values of the mtDNA genes being 3.5-fold times higher than for the ptDNA genes. The nonsynonymous substitutions for different species within the same genome were very similar (Table 2). The average nonsynonymous substitution (dN) values for all of the species varied from 0.04 to 0.05 for the mitochondrial genes, and from 0.02 to 0.03 for the chloroplast genes. When we compared the dN values between the mitochondrial genes and chloroplast genes, we also found that all of the species showed higher average dN values in mtDNA. Specifically, the values for the mtDNA genes were 1.33- to 2.67-fold higher than the ptDNA genes. A different pattern was observed for the dN/dS ratio (Table 2). For instance, only three species (*Chlamydomonas reinhardtii*, *Gonium pectorale*, and *Pandorina colemaniae*) showed higher dN/dS ratios in mtDNA. All of the other species had lower dN/dS ratios in mtDNA. However, the dN/dS ratios between the different genomes were very similar, and did not vary by more than two-fold.

**Figure 4:**
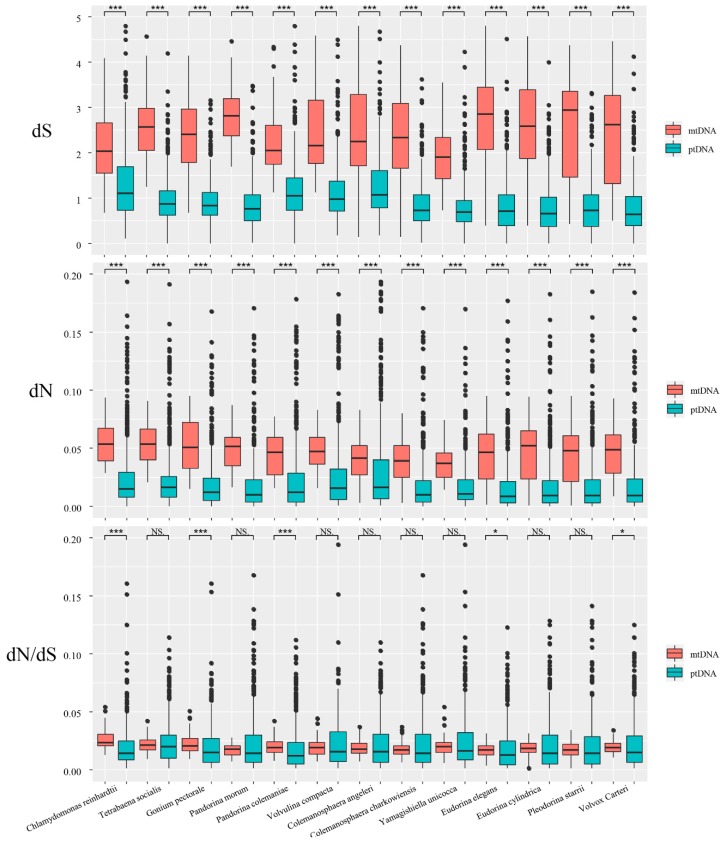
Boxplots showing synonymous substitutions (dS), nonsynonymous substitutions (dN), and dN/dS ratios in mtDNA and ptDNA among different volvocine algae. The box represents the values between the quartiles. Outliers are shown as black points, and the black lines inside the box represent the median values. (***p < 0.001, ** *p* < 0.01, * *p* < 0.05, and NS—not significant).

We compared the substitution rates and dN/dS ratios between mtDNA and ptDNA in all of the species using the Wilcoxon rank sum test (Figure 4). All of the species showed significantly higher dN and dS values for mtDNA compared with ptDNA (*p* < 0.001). Regarding the dN/dS ratio, only five species (*Chlamydomonas reinhardtii, Gonium pectorale, Pandorina colemaniae, Eudorina elegans,* and *Volvox carteri*) showed significant differences between mtDNA and ptDNA.

## 4. Discussion

### 4.1. Phylogenetic Analysis

In this study, we found phylogenetic incongruence between supermatrix and coalescent-based analyses. We also found phylogenetic incongruence between our results and previous studies. Thus, a portion of our phylogenetic results could not reflect the true evolutionary relationship between colonial volvocine algae. There are three main reasons for phylogenetic incongruence, namely: systematic error, stochastic error, and biological factors.

The reconstruction method cannot completely account for the properties of the data that would lead to systematic errors [53]. However, the LogDet distances, RY-coding strategy, and covarion model showed that a systematic error was not severe enough to affect the phylogenetic results of the genus *Yamagishiella* in our study. The stochastic error in the phylogenetic construction was caused by the limited length of the sequence used in the inference [53]. As we only discussed the validity of phylogenetic construction based on mtDNA protein coding genes in this study, and the total length of our concatenated dataset was only 5382 nt, our results may thus be affected by a stochastic error. Moreover, phylogenetic analysis based on unlinked loci is an appropriate way to reduce phylogenetic incongruence [54], but all of the genes we used are located in the mitochondrial genome, which does not combine with other genomes thus forming a single linkage group. Thus, the phylogenetic results of our study should only be considered merely as gene trees [55]. Here, we suspect that the stochastic error and linkage group we used may be partly responsible for the phylogenetic incongruence of the genus *Yamagishiella* between our study and previous studies.

The supermatrix and coalescent-based analyses showed phylogenetic incongruence in the genus *Colemanosphaera*. Our coalescent-based analysis for the genus *Colemanosphaera* is consistent with Nozaki et al. [23], but this phylogenetic relationship is not supported by the supermatrix approach. This incongruence may result from the different reconstruction approach. The supermatrix approach is the most accurate species tree reconstruction approach when incomplete lineage sorting (ILS) and horizontal gene transfer (HGT) are low [56,57,58], but this approach is less robust than coalescent-based approaches under high levels of ILS and HGT [59,60,61]. Here, we suspect that the mtDNA protein-coding genes may not reflect the true species tree as a result of biological factors (i.e., ILS and HGT).

In this study, we also found phylogenetic incongruence in the clades *Eudorina*/*Pleodorina*/*Volvox* and *Pandorina*/*Volvulina*. The genera in these two clades are all polyphyletic [62,63], and we only used a limited number of species. Thus, their incongruence will be assessed in future studies with more related species.

### 4.2. Substitution Rate Estimation

Drouin et al. [28] investigated the substitution rates in seed plants, and found that all seed plants showed higher substitution rates for ptDNA compared with mtDNA. However, Smith [26] analyzed the substitution rates in diverse lineages, and suggested that the opposite is true in algae. The results of our study are consistent with the results of Smith [26]. We also found that mtDNA showed a higher substitution rate than ptDNA. Two explanations were put forward to illustrate this phenomenon, as follows: the fidelity and efficacy of mitochondrial maintenance machineries are more variable than those of ptDNA, and fewer mtDNA genes allow for larger fluctuations in the mutation rate [26].

We also analyzed the dN/dS ratio. Most species showed no significant difference between mtDNA and ptDNA, indicating that protein-coding genes in the mitochondrial and chloroplast genomes are under similar selection pressure. Five species showed significant differences (*p* < 0.05) between mtDNA and ptDNA, but these species have similar average values, median values, and quartiles for the two counterparts (Table 2 and Figure 4). We noticed that ptDNA has more outliers (higher than upper quartiles) than mtDNA (Figure 4). In these five species, we suspect that the limited number of ptDNA genes with high dN/dS ratios may be the main factor causing a significant difference between mtDNA and ptDNA.

## 5. Conclusions

In this study, we present six newly sequenced mitochondrial genomes of colonial volvocine algae. Our phylogenetic analysis showed that mitochondrial protein-coding genes are not suitable for reconstructing the phylogenetic relationship among colonial volvocine algae, given that the phylogenetic result may be affected by systematic errors, stochastic errors, and biological factors. We also analyzed the substitution rates of ptDNA and mtDNA among colonial volvocine algae. The results suggest that all species have higher mtDNA substitution rates compared with ptDNA. The dN/dS ratio is similar between ptDNA and mtDNA in most colonial volvocine species, indicating a similar selection pressure in the two counterparts. However, few ptDNA protein-coding genes have high dN/dS ratios, resulting in a significant difference between ptDNA and mtDNA in five colonial volvocine species.

## Figures and Tables

**Table 1:** Mitochondrial genome features of colonial volvocine algae. The species sequenced in this study are indicated in bold.

Species	Cell Number ^a^	Size (kb)	GC Content	Number of Scaffold	Number of Genes			Genbank Accession
					Protein-Coding Genes ^b^	rRNA Genes	tRNA Genes	
*Tetrabaena socialis*	4	28	45%	1	9	12	3	KX232644
*Gonium pectorale*	8–32	16	39%	1	8	12	4	AP012493
***Pandorina morum***	8–16	15	45%	5	7	3	1	MN431468-MN431472
***Pandorina colemaniae***	8–16	19	38%	1	8	9	3	MN431473
***Volvulina compacta***	8–16	22	41%	1	8	6	4	MN431476
***Colemanosphaera angeleri***	16–32	18	38%	1	10	8	3	MN431475
***Colemanosphaera charkowiensis***	16–32	24	38%	1	9	8	3	MN431474
***Yamagishiella unicocca***	16–32	24	41%	1	9	12	3	MN431477
*Yamagishiella unicocca*	16–32	31	41%	1	12	12	3	KY442293
*Eudorina elegans*	16–32	27	33%	1	11	12	3	MH161346
*Eudorina cylindrica*	16–32	26	36%	1	9	12	3	MH223465
*Eudorina* sp.	16–32	21	38%	1	9	12	3	KY442294
*Pleodorina starrii*	32–128	20	38%	1	10	12	3	JX977845
*Volvox carteri*	500–50,000	35	34%	1	10	13	3	GU084821

^a^ According to Wehr et al. [46]; ^b^ protein-coding genes include open reading frame (ORF) genes.

**Table 2:** Chloroplast and mitochondrial DNA substitution rates in the colonial volvocine algae. Abbreviations are as follows: chloroplast genome (ptDNA), mitochondrial genome (mtDNA), mt:pt is the ratio of value from mtDNA to value from ptDNA.

Species	Synonymous Substitution (dS)			Nonsynonymous Substitution (dN)			dN/dS		
	mtDNA	ptDNA	mt:pt	mtDNA	ptDNA	mt:pt	mtDNA	ptDNA	mt:pt
*Chlamydomonas reinhardtii*	2.2 ± 0.87	1.31 ± 0.83	1.8:1	0.05 ± 0.02	0.03 ± 0.03	2.17:1	0.03 ± 0.01	0.02 ± 0.02	1.36:1
*Tetrabaena socialis*	2.57 ± 0.73	0.95 ± 0.52	2.74:1	0.05 ± 0.02	0.02 ± 0.03	2.33:1	0.02 ± 0.01	0.02 ± 0.03	0.88:1
*Gonium pectorale*	2.41 ± 0.86	0.93 ± 0.51	2.84:1	0.05 ± 0.02	0.02 ± 0.02	2.67:1	0.02 ± 0.01	0.02 ± 0.02	1.12:1
*Pandorina morum*	2.85 ± 0.66	0.82 ± 0.51	3.5:1	0.05 ± 0.02	0.02 ± 0.03	2.65:1	0.02 ± 0.01	0.02 ± 0.03	0.78:1
*Pandorina colemaniae*	2.25 ± 0.76	1.14 ± 0.65	2.07:1	0.04 ± 0.02	0.02 ± 0.03	1.92:1	0.02 ± 0.01	0.02 ± 0.02	1.1:1
*Volvulina compacta*	2.54 ± 0.92	1.13 ± 0.64	2.39:1	0.05 ± 0.02	0.03 ± 0.03	1.85:1	0.02 ± 0.01	0.02 ± 0.02	0.96:1
*Colemanosphaera angeleri*	2.38 ± 1.13	1.24 ± 0.69	1.95:1	0.04 ± 0.02	0.03 ± 0.04	1.33:1	0.02 ± 0.01	0.02 ± 0.02	0.87:1
*Colemanosphaera charkowiensis*	2.35 ± 1.11	0.82 ± 0.54	2.96:1	0.04 ± 0.02	0.02 ± 0.03	2.11:1	0.02 ± 0.01	0.02 ± 0.03	0.81:1
*Yamagishiella unicocca*	1.97 ± 0.72	0.8 ± 0.52	2.55:1	0.04 ± 0.02	0.02 ± 0.02	2.03:1	0.02 ± 0.01	0.02 ± 0.03	0.81:1
*Eudorina elegans*	2.57 ± 1.22	0.83 ± 0.61	3.1:1	0.04 ± 0.03	0.02 ± 0.02	2.62:1	0.02 ± 0.01	0.02 ± 0.02	0.93:1
*Eudorina cylindrica*	2.49 ± 1.24	0.78 ± 0.55	3.37:1	0.05 ± 0.03	0.02 ± 0.02	2.64:1	0.02 ± 0.01	0.02 ± 0.02	0.86:1
*Pleodorina starrii*	2.51 ± 1.25	0.81 ± 0.57	3:1	0.04 ± 0.03	0.02 ± 0.03	2.44:1	0.02 ± 0.01	0.02 ± 0.03	0.79:1
*Volvox carteri*	2.42 ± 1.18	0.78 ± 0.56	3.21:1	0.05 ± 0.02	0.02 ± 0.02	2.58:1	0.02 ± 0.01	0.02 ± 0.02	0.91:1

## References

[1] Moore L.J., Coleman A.W. (1989). The linear 20 kb mitochondrial genome of *Pandorina morum* (Volvocaceae, Chlorophyta). Plant Mol. Biol..

[2] Smith D.R., Lee R.W. (2010). Low nucleotide diversity for the expanded organelle and nuclear genomes of *Volvox carteri* supports the mutational-hazard hypothesis. Mol. Biol. Evol..

[3] Hamaji T., Smith D.R., Noguchi H., Toyoda A., Suzuki M., Kawai-Toyooka H., Fujiyama A., Nishii I., Marriage T., Olson O. (2013). Mitochondrial and plastid genomes of the colonial green alga *Gonium pectorale* give insights into the origins of organelle DNA architecture within the Volvocales. PLoS ONE.

[4] Smith D.R., Hamaji T., Olson B.J., Durand P.M., Ferris P., Michod R.E., Featherston J., Nozaki H., Keeling P.J. (2013). Organelle genome complexity scales positively with organism size in volvocine green algae. Mol. Biol. Evol..

[5] Featherston J., Arakaki Y., Nozaki H., Durand P.M., Smith D.R. (2016). Inflated organelle genomes and a circular-mapping mtDNA probably existed at the origin of coloniality in volvocine green algae. Eur. J. Phycol..

[6] Hamaji T., Kawai-Toyooka H., Toyoda A., Minakuchi Y., Suzuki M., Fujiyama A., Nozaki H., Smith D.R. (2017). Multiple independent changes in mitochondrial genome conformation in Chlamydomonadalean algae. Genome Biol. Evol..

[7] Hu Y., Xing W., Song H., Liu G., Hu Z. (2019). Analysis of mitochondrial and chloroplast genomes in two volvocine algae: *Eudorina elegans* and *Eudorina cylindrica* (Volvocaceae, Chlorophyta). Eur. J. Phycol..

[8] Hall J.D., Karol K.G., McCourt R.M., Delwiche C.F. (2008). Phylogeny of the conjugating green algae based on chloroplast and mitochondrial nucleotide sequence data. J. Phycol..

[9] Durand J.D., Borsa P. (2015). Mitochondrial phylogeny of grey mullets (Acanthopterygii: Mugilidae) suggests high proportion of cryptic species. Comptes Rendus Biol..

[10] Lee J.M., Song H.J., Park S.I., Lee Y.M., Jeong S.Y., Cho T.O., Kim J.H., Choi H., Choi C.G., Nelson W.A. (2018). Mitochondrial and plastid genomes from coralline red algae provide insights into the incongruent evolutionary histories of organelles. Genome Biol. Evol..

[11] Vanhove M.P., Briscoe A.G., Jorissen M.W., Littlewood D.T.J., Huyse T. (2018). The first next-generation sequencing approach to the mitochondrial phylogeny of African monogenean parasites (Platyhelminthes: Gyrodactylidae and Dactylogyridae). BMC Genom..

[12] Sousa C.B., Cox C.J., Brito L., Pavão M.M., Pereira H., Ferreira A., Ginja C., Campino L., Bermejo R., Parente M. (2019). Improved phylogeny of brown algae *Cystoseira* (Fucales) from the Atlantic-Mediterranean region based on mitochondrial sequences. PLoS ONE.

[13] Angeler D.G., Schagerl M., Coleman A.W. (1999). Phylogenetic relationships among isolates of *Eudorina* species (Volvocales, Chlorophyta) inferred from molecular and biochemical data. J. Phycol..

[14] Coleman A.W. (2001). Biogeography and speciation in the *Pandorina*/*Volvulina* (Chlorophyta) superclade. J. Phycol..

[15] Yamada T.K., Miyaji K., Nozaki H. (2008). A taxonomic study of *Eudorina unicocca* (Volvocaceae, Chlorophyceae) and related species, based on morphology and molecular phylogeny. Eur. J. Phycol..

[16] Hayama M., Nakada T., Hamaji T., Nozaki H. (2010). Morphology, molecular phylogeny and taxonomy of *Gonium maiaprilis* sp. nov. (Goniaceae, Chlorophyta) from Japan. Phycologia.

[17] Nakada T., Tomita M., Nozaki H. (2010). *Volvulina compacta* (Volvocaceae, Chlorophyceae), new to Japan, and its phylogenetic position. J. Jpn. Bot..

[18] Nozaki H., Ito M., Sano R., Uchida H., Watanabe M.M., Takahashi H., Kuroiwa T. (1997). Phylogenetic analysis of *Yamagishiella* and *Platydorina* (Volvocaceae, Chlorophyta) based on *rbcL* gene sequences. J. Phycol..

[19] Nozaki H., Ito M., Uchida H., Watanabe M.M., Kuroiwa T. (1997). Phylogenetic analysis of *Eudorina* species (Volvocaceae, Chlorophyta) based on *rbcL* gene sequences. J. Phycol..

[20] Nozaki H., Song L., Liu Y., Hiroki M., Watanabe M.M. (1998). Taxonomic re-examination of a Chinese strain labeled ‘*Eudorina* sp.’ (Volvocaceae, Chlorophyta) based on morphological and DNA sequence data. Phycol. Res..

[21] Nozaki H., Misawa K., Kajita T., Kato M., Nohara S., Watanabe M.M. (2000). Origin and evolution of the colonial Volvocales (Chlorophyceae) as inferred from multiple, chloroplast gene sequences. Mol. Phylogenet. Evol..

[22] Nozaki H., Ott F.D., Coleman A.W. (2006). Morphology, molecular phylogeny and taxonomy of two new species of *Pleodorina* (Volvoceae, Chlorophyceae). J. Phycol..

[23] Nozaki H., Yamada T.K., Takahashi F., Matsuzaki R., Nakada T. (2014). New “missing link” genus of the colonial volvocine green algae gives insights into the evolution of oogamy. BMC Evol. Biol..

[24] Nozaki H., Matsuzaki R., Yamamoto K., Kawachi M., Takahashi F. (2015). Delineating a new heterothallic species of *Volvox* (Volvocaceae, Chlorophyceae) using new strains of “*Volvox africanus*”. PLoS ONE.

[25] Hu Y., Xing W., Song H., Zhu H., Liu G., Hu Z. (2019). Evolutionary analysis of unicellular species in Chlamydomonadales through chloroplast genome comparison with the colonial volvocine algae. Front. Microbiol..

[26] Smith D.R. (2015). Mutation rates in plastid genomes: They are lower than you might think. Genome Biol. Evol..

[27] Brown W.M., George M., Wilson A.C. (1979). Rapid evolution of animal mitochondrial DNA. Proc. Natl. Acad. Sci. USA.

[28] Drouin G., Daoud H., Xia J. (2008). Relative rates of synonymous substitutions in the mitochondrial, chloroplast and nuclear genomes of seed plants. Mol. Phylogenet. Evol..

[29] Smith D.R., Arrigo K.R., Alderkamp A.C., Allen A.E. (2014). Massive difference in synonymous substitution rates among mitochondrial, plastid, and nuclear genes of Phaeocystis algae. Mol. Phylogenet. Evol..

[30] Grisdale C.J., Smith D.R., Archibald J.M. (2019). Relative mutation rates in nucleomorph-bearing algae. Genome Biol. Evol..

[31] Sloan D.B., Taylor D.R. (2012). Evolutionary rate variation in organelle genomes: The role of mutational processes. Organelle Genetics.

[32] Bernt M., Donath A., Jühling F., Externbrink F., Florentz C., Fritzsch G., PÜTZ J., Stadler P.F. (2013). MITOS: Improved de novo metazoan mitochondrial genome annotation. Mol. Phylogenet. Evol..

[33] Camacho C., Coulouris G., Avagyan V., Ma N., Papadopoulos J., Bealer K., Madden T.L. (2009). BLAST+: Architecture and applications. BMC Bioinf..

[34] Lowe T.M., Chan P.P. (2016). tRNAscan-SE On-line: Integrating search and context for analysis of transfer RNA genes. Nucleic Acids Res..

[35] Darling A.E., Mau B., Perna N.T. (2010). progressiveMauve: Multiple genome alignment with gene gain, loss and rearrangement. PLoS ONE.

[36] Katoh K., Standley D.M. (2013). MAFFT multiple sequence alignment software version 7, Improvements in performance and usability. Mol. Biol. Evol..

[37] Abascal F., Zardoya R., Telford M.J. (2010). TranslatorX: Multiple alignment of nucleotide sequences guided by amino acid translations. Nucleic Acids Res..

[38] Capella-Gutiérrez S., Silla-Martínez J.M., Gabaldón T. (2009). trimAl: A tool for automated alignment trimming in large-scale phylogenetic analyses. Bioinformatics.

[39] Smith S.A., Dunn C.W. (2008). Phyutility: A phyloinformatics tool for trees, alignments and molecular data. Bioinformatics.

[40] Lanfear R., Frandsen P.B., Wright A.M., Senfeld T., Calcott B. (2016). PartitionFinder 2, New methods for selecting partitioned models of evolution for molecular and morphological phylogenetic analyses. Mol. Biol. Evol..

[41] Stamatakis A. (2014). RAxML version 8, A tool for phylogenetic analysis and post-analysis of large phylogenies. Bioinformatics.

[42] Ronquist F., Teslenko M., Mark P.V.D., Ayres D.L., Darling A., Höhna S., Larget B., Liu L., Suchard M.A., Huelsenbeck J.P. (2012). MrBayes 3.2, Efficient Bayesian phylogenetic inference and model choice across a large model space. Syst. Biol..

[43] Zhang C., Rabiee M., Sayyari E., Mirarab S. (2018). ASTRAL-III: Polynomial time species tree reconstruction from partially resolved gene trees. BMC Bioinf..

[44] Phillips M.J., Delsuc F., Penny D. (2004). Genome-scale phylogeny and the detection of systematic biases. Mol. Biol. Evol..

[45] Yang Z. (2007). PAML 4, Phylogenetic analysis by maximum likelihood. Mol. Biol. Evol..

[46] Wehr J.D., Sheath R.G., Kociolek J.P. (2015). Freshwater Algae of North America: Ecology and Classification.

[47] Bleidorn C. (2017). Sources of error and incongruence in phylogenomic analyses. Phylogenomics.

[48] Jeffroy O., Brinkmann H., Delsuc F., Philippe H. (2006). Phylogenomics: The beginning of incongruence?. Trends Genet..

[49] Lockhart P.J., Steel M.A., Hendy M.D., Penny D. (1994). Recovering evolutionary trees under a more realistic model of sequence evolution. Mol. Biol. Evol..

[50] Woese C.R., Achenbach L., Rouviere P., Mandelco L. (1991). Archaeal phylogeny: Reexamination of the phylogenetic position of *Archaeoglohus fulgidus* in light of certain composition-induced artifacts. Syst. Appl. Microbiol..

[51] Huelsenbeck J.P. (2002). Testing a covariotide model of DNA substitution. Mol. Biol. Evol..

[52] Yang Z., Nielsen R. (2000). Estimating synonymous and nonsynonymous substitution rates under realistic evolutionary models. Mol. Biol. Evol..

[53] Delsuc F., Brinkmann H., Philippe H. (2005). Phylogenomics and the reconstruction of the tree of life. Nat. Rev. Genet..

[54] Hu Y., Xing W., Song H., Hu Z., Liu G. (2019). Comparison of colonial volvocine algae based on phylotranscriptomic analysis of gene family evolution and natural selection. Eur. J. Phycol..

[55] Sang T. (2002). Utility of low-copy nuclear gene sequences in plant phylogenetics. Crit. Rev. Biochem. Mol. Biol..

[56] Mirarab S., Bayzid M.S., Warnow T. (2014). Evaluating summary methods for multilocus species tree estimation in the presence of incomplete lineage sorting. Syst. Biol..

[57] Bayzid M.S., Mirarab S., Boussau B., Warnow T. (2015). Weighted statistical binning: Enabling statistically consistent genome-scale phylogenetic analyses. PLoS ONE.

[58] Mallo D., Posada D. (2016). Multilocus inference of species trees and DNA barcoding. Philos. Trans. R. Soc. B.

[59] Mirarab S., Reaz R., Bayzid M.S., Zimmermann T., Swenson M.S., Warnow T. (2014). ASTRAL: Genome-scale coalescent-based species tree estimation. Bioinformatics.

[60] Davidson R., Vachaspati P., Mirarab S., Warnow T. (2015). Phylogenomic species tree estimation in the presence of incomplete lineage sorting and horizontal gene transfer. BMC Genom..

[61] Mirarab S. (2019). Species Tree Estimation Using ASTRAL: Practical Considerations. arXiv.

[62] Hanschen E.R., Herron M.D., Wiens J.J., Nozaki H., Michod R.E. (2018). Repeated evolution and reversibility of self-fertilization in the volvocine green algae. Evolution.

[63] Umen J., Coelho S. (2019). Algal Sex Determination and the Evolution of Anisogamy. Annu. Rev. Microbiol..

